# Everyday Healthcare Regulation: British Newspapers and Complementary and Alternative Medicine

**DOI:** 10.1177/09646639231211234

**Published:** 2023-11-09

**Authors:** Michael Ashworth

**Affiliations:** University of Newcastle Law School, Newcastle upon Tyne, UK

**Keywords:** complementary and alternative medicine, decentred regulation, mass media, news

## Abstract

This article interrogates the controversial field of complementary and alternative medicine (CAM), focussing in particular on the implication of the British press in its regulation. It grounds its analysis in a ‘decentred’ understanding of regulation; a socio-legal approach which moves beyond formal regulation and regulators, and instead foregrounds diverse social actors and their attempts to alter behaviour across a given domain. Focussing on *The Times* newspaper as a case-study, it identifies five regulatory techniques through which the newspaper drew (and redrew) lines separating the safe from the risky, the efficacious from the sham, and the normal from the deviant. By analytically decentring CAM's formal regulation, this article provides a conceptual contribution. It highlights an everyday form of healthcare regulation directed at prospective users which may be just as significant in potentially guiding users towards or away from particular healthcare practices/practitioners as the more traditional, formal kinds of regulation identified in regulatory literatures.

## Introduction

This article interrogates the controversial field of complementary and alternative medicine (CAM), focussing in particular on the implication of the British press in its regulation. In so doing, it builds upon a ‘decentred’ understanding of regulation, which moves beyond a strict focus on formal regulation/regulators, and instead foregrounds diverse social agents and their attempts, intentional or otherwise, to alter the behaviour of actors (Baldwin et al., 2012; Black, 2002). Regulatory literatures on CAM in the UK have thus far generally expounded on both the heterogeneity of CAM, underpinned by diverse epistemologies of healing and illness, and on the complexity of the formal regulatory regime surrounding it (Treweek et al., 2019). This formal regime encompasses a relatively smaller number of medically qualified CAM practitioners, such as doctor-homeopaths, statutorily regulated by the General Medical Council (GMC), working within and outside of the publicly funded National Health Service (NHS); and, a much larger cadre of nonmedically qualified (NMQ) healers, operating overwhelmingly in the private sphere, whose regulation is mostly ‘voluntary’^1^ and consists of somewhat fragmented layers of registers, private training courses of different lengths, professional associations underpinned by variable standards, umbrella bodies and CAM councils (Cant and Sharma, 1996; Saks, 1999).

However, as decentred regulatory scholarship in other fields has demonstrated, formal regulation, whether state-enforced or ‘voluntary’, is only part of the regulatory story (Drahos and Krygier, 2017). In a move reminiscent of the decentring of (formal) law enacted by legal pluralists (Kingsford Smith, 2002; Merry, 1988), the decentring of regulation has opened up the conceptual imaginary of who counts as a regulator/regulatee and what constitutes regulation (Healy, 2017). Building on the space opened up by such scholarship, this article analytically decentres CAM's formal regulation, broadening the regulatory scope to include the news media. While recognised in the literature as displaying a strong appetite for stories on CAM over the years (Weeks and Strudsholm, 2008), the British news media have not yet themselves been conceptualised as exercising regulatory power over CAM. By focussing on the role of daily newspapers – and using *The Times* newspaper as a specific case study – this article makes an original conceptual contribution, bringing into sharp relief a practical, ‘everyday’ form of healthcare regulation; one which operates primarily through guiding (or, rather, attempting to guide) the self-regulatory capacity of health consumers. Moreover, owing to the news media's everyday nature, this form of regulation may be just as significant in potentially altering behaviour when it comes to selecting CAMs (or staying away from them) as the more formal conceptions of regulation found in the literature.

The article is structured as follows. First, I provide the key context, outlining what CAM is and how it has been formally regulated in the UK, before moving on to explore the literature on decentred regulation and its application in healthcare contexts. In the following section, on methods, I contextualise *The Times* newspaper within the broader UK media landscape and describe my analytical approach to the dataset, covering 1970 to 2019. In analysing the newspaper data, I identify five regulatory techniques through which prospective CAM users’ behaviour was potentially guided: normalisation of CAMs by biomedicine; problematisation of biomedicine; highlighting risks associated with poor practice; auditing practitioners and ranking CAM therapies; and, (re)problematising CAMs. In so doing, I demonstrate that the coverage seemingly shares some of the functions and concerns typically associated with more formal healthcare regulators – namely, attempting to ensure patients access safe and/or efficacious forms of healing from competent practitioners (however safety, efficacy and competency are defined). Moreover, in the coverage, I demonstrate that the purported quality of existing formal regulatory regimes is itself one of the means by which users are guided towards or away from CAMs and their professionals. In the conclusion, I consider the broader implications of the circulation of such everyday healthcare regulation through the mass media in relation to other aspects of healthcare and public health, such as the COVID-19 pandemic.

## Contextualising CAM and its Regulation in the UK

The umbrella term CAM refers to a diverse field of practices, philosophies and approaches to ill-health and healing, which ‘vary from one movement or tradition to another and form no consistent … body of knowledge’ (Gevitz, 1995). Indeed, it appears that CAM can only be unified in the sense that it is not biomedicine; which refers to biological theories of disease and ill-health, uses surgery and pharmaceuticals as its primary therapeutic instruments, and is often described as treating the body as a machine to be fixed. By contrast, CAM therapies often operate on a different epistemological basis, encompassing alternative ways of seeing and knowing illness and inducing healing (Fulder, 1998). CAM draws on a broader range of diagnostic and therapeutic categories than biomedicine, ranging from the bodily and the physiological, through to the psychic, spiritual or cosmological (Kaptchuk and Eisenberg, 2005).

Not only is CAM's epistemologically plural nature a source of controversy, with frequent accusations of quackery owing to its ‘unproven’ or ‘implausible’ claims (British Medical Association, 1986) but, as medical sociology, science and technology studies and recent socio-legal scholarship has demonstrated, it generates tensions when formal regulations attempt to order it, particularly when law and state are involved (Adams, 2003; Janes, 1999; Pordié, 2010). This is because, from the perspective of CAM's critics, regulation is seen as legitimising a fundamentally illegitimate field of practice (Wahlberg, 2015). From the perspective of CAM practitioners, however, tensions arise because the tools law has at its disposal to enact its regulatory tidying up are the very tools of biomedicine (Ashworth and Cloatre, 2022; Cloatre, 2019); a significant problem for those CAM therapies unable to ‘prove’ themselves according to the tests valued by biomedical science, such as the randomised controlled trial (RTC) (Urquiza-Haas and Cloatre, 2021).

In the UK, CAM has been formally regulated through what the literature describes as ‘soft’, ‘light-touch’, ‘market-based’ or ‘voluntary’ self-regulatory mechanisms. These are terms which are generally counterposed to top-down, legislative ‘command and control’ regulation (Baldwin et al., 1998), insofar as they describe a situation in which ‘the body that promulgates the regulation is, at the same time, subjected to it’ (De Minico, 2006: 188). Such self-regulation entails ‘no binding rules, no role for courts, no forcible implementation by the state’, and often, ‘no public agency at all’ (Töller, 2011: 499). Rather than altering behaviour through deterrence and punishment enforced by law and state, they induce (or attempt to induce) adherence to formal standards, codes of conduct, informational strategies and so on, through normative soft power.

Myriad voluntary registers and practitioners’ associations for specific therapies, groups of therapies and/or CAM as a whole have emerged since the 1980s in the UK (Saks, 1999). There has been, at times, considerable regulatory fragmentation, with multiple bodies claiming to represent and regulate a given CAM therapy and its practitioners (Stone and Lee-Treweek, 2005). Much of the regulatory literature has attended to this formal regulatory regime and highlighted key moments in governmental debates over the nature of CAM's regulation – in particular, whether statutory self-regulation (SSR) might be achievable. Such moments have included: generalised calls in Parliament for healers to ‘get their own house in order’ in the mid-1980s as a pre-requisite for achieving their goals of SSR (Wahlberg, 2015); a Parliamentary report in 2000, which supported SSR for acupuncture, herbalism and homeopathy (the most credible and professionally organised of the ‘unregulated’ CAM therapies, per the report) but laid to rest the SSR ambitions of other fields of practice, instead calling on them to unify behind a single voluntary regulator for each CAM discipline (Wahlberg, 2007); and, most recently, in 2012, the emergence of the Professional Standards Authority (PSA) – an independent meta-regulator of the statutorily regulated health professions – along with its voluntary accreditation scheme for (market-based) registers of ‘unregulated’ health professionals, such as registers of CAM therapies (Stone, 2015).

Turning to decentred regulatory scholarship, however, it appears that formal regulators/regulations are only part of the regulatory story – and perhaps one that ought not to be necessarily privileged in analyses. This is because decentred regulation scholarship, with its diverse frameworks and areas of focus, has not only conceptually shifted scholarly attention away from the law and state but, more radically, away from ‘even well recognised forms of self-regulation’ (Black, 2002: 1). Fixed notions of who counts as regulator and regulatee have been shaken, as regulation has come to be conceptualised by some, in a manner influenced by Foucauldian understandings of power, as capillary, dispersed across society, as something which quite simply ‘happens’ through interactions of social actors, sometimes even regardless of subjective intentions (Black, 2002). By broadening the analytical lens, the regulatory activities of different actors, including credit agencies (Scott, 2002), housing associations (Cowan and McDermont, 2006) and even prisoners (Buck and Tomczak, 2022), have been brought to the fore. Such a radical decentring therefore opens up new sites, actors and objects for regulatory exploration, bringing scholarly attention to potentially significant means of influencing behaviour that would otherwise have been occluded through a focus on more traditional, formal kinds of regulation.

In the field of health, decentred regulatory approaches drawn from different national contexts have shone a light on the regulatory activities of diverse actors and techniques, particularly in the field of in private healthcare. Hunter et al. (2022), in exploring private healthcare in India, consider the regulation exercised by actors such as insurance companies, commercial systems of accreditation, and technological intermediaries which provide information for prospective healthcare users on pricing and user-generated ratings of health facilities. Trubek et al. (2008), writing predominantly about the US and Europe, similarly highlight the regulatory role of commercial organisations, their production and monitoring of standards, and the use of technological tools directed towards the health consumer. Indeed, the prospective healthcare user looms large in some decentred regulatory literatures, to the extent that some go as far as inverting traditional conceptualisations of regulatory relationships by suggesting that patients themselves may be regulators of healthcare (Healy, 2017).

If such literatures have highlighted the ‘polycentric’ nature of healthcare regulation and, in so doing, underscored the healthcare user as a significant regulatory actor (Dixon-Woods, 2019: 53), it is perhaps surprising that the news media has not featured significantly in decentred scholarship. To be sure, some have gestured towards the potential of the media as a regulatory actor for biomedical healthcare, through activities such as publicising poor professional practice (thereby triggering enforcement action from formal regulators) or, in the case of private healthcare, ranking providers so users may choose ‘higher-quality’ services (Ensor and Weinzierl, 2007). Yet such an analytical decentring, within or outside the media, has yet to significantly occur in relation to CAM. This is particularly notable, given that CAM in the UK is predominantly a private form of healthcare that has received extensive media coverage (Doel and Segrott, 2003b; Heller and Stone, 2005; Weeks and Strudsholm, 2008), while at the same time, centralised information from state-authorised sources about CAM have, for the most part, been absent (House of Lords, 2000).

This article reconceptualises news media as a broad site of decentred regulation and, focussing on daily newspapers in particular, it interrogates some of the regulatory techniques employed in the coverage of CAM. That is, it explores the means by which such news media have potentially influenced readers/prospective CAM users towards or away from CAMs and their professionals on the basis of distinguishing the safe from the risky, the effective from the sham, and the normal from the deviant.

## Methods

This article focuses on data drawn from *The Times* newspaper – a centre-right broadsheet privately owned since 1981 by the international media conglomerate News Corp. *The Times* is part of a diverse UK media landscape which comprises: print (often sub-divided between so-called ‘quality’ titles, including *The Times* and *The Guardian*, or tabloids, including *The Sun* and *Daily Mirror*), broadcast (television and radio outlets, such as the publicly funded BBC News and privately owned competitors, such as Sky), digital (including high-traffic news websites such as BBC News Online, The Mail Online, or The Guardian) and social media (e.g. TikTok, Instagram, Twitter and Facebook, which provide algorithmic platforms for the spread of ‘official’ sources of information, from news organisations and professional journalists, alongside more ‘unofficial’ sources, such as citizen journalists, members of the public, and so on). The use of print news as the basis of this article might therefore be seen as ‘old school’, given declining print circulation figures – and the corresponding ascendence of digital news and social media. Indeed, *The Times* hit a print circulation peak of 726,349 in 2000; a number which had dipped to 417,298 by 2019 (Historic Newspapers, 2021). However, print and other forms of media are not mutually exclusive: news stories which appear in print also appear across other forms of media, while titles such as *The Times* maintain a highly visible presence across digital and social media platforms. More importantly, given the historical breadth of the dataset (1 January 1970 to 31 December 2019), for much of which print news predominated, I prioritised comparing and analysing like with like, teasing out the kinds of techniques evident across the same types of data: front pages, ‘hard’ new articles, editorials, features, opinion editorials/columns, and, specialist supplements.

*The Times* newspaper was selected as the case study for several reasons. First, due to its typical readership – older, predominantly ‘ABC1’ or middle class – which tends to overlap with the demographic most likely to use CAM in the UK, given that it is primarily an out-of-pocket form of healthcare which is accessible to those who can afford to pay (Loudon, 2006). Second, due to its historic reputation as the ‘newspaper of record’, a reputation which implies influence beyond its immediate readership and a certain establishment credibility, detached seriousness and even neutrality (which belies its ideological bent). Its reputation and influence are particularly evident through its tendency to publish ‘authoritative’ guides, league tables and rankings on different issues – most famously, *The Times Good University Guide*, published since the early 1990s. Finally, and more practically, it was chosen due to the accessibility and comprehensiveness of its print archives, which stretches from the required start date of 1970 through to the end of 2019, albeit with a gap of 1 year between 1978 and 1979 due to a labour strike in which production was shutdown.

I performed keyword searches for CAM and prolific practices via the Gale Primary News Database between 1 January 1970 (the decade when CAM usage accelerated in the UK) and the end of 2019 (where Gale's database for *The Times* ends), resulting in a dataset comprising 792 items. Adopting a theoretically informed deductive analytical strategy, I coded the data through the lens of a Foucauldian-inflected decentred regulatory perspective, meaning my focus did not lie in building up a quantitative picture to ascertain shifting trends. Rather, I sought to qualitatively highlight aspects of the data illustrative of the kinds of techniques which may have influenced reader behaviour in relation to the safety, efficacy and/or quality of CAMs. Through the analysis it became apparent that different techniques appeared alongside others to varying degrees, rather than forming discrete regulatory episodes. Nevertheless, I qualitatively noted the relative intensity of such techniques, as they waxed and waned throughout the dataset, and included this within the analysis where appropriate.

Finally, it should be clarified that, as much of the regulatory literature highlights, the existence of regulation is not to be confused with the question of its effectiveness. While this article makes a conceptual contribution in highlighting an informal regulatory regime in the media, it makes no comment on the extent to which it was effective in guiding the conduct of prospective CAM users: that is an empirical line of inquiry which this article leaves open for future scholarship. Additionally, that the news media can be conceptualised as a domain of decentred regulation is not necessarily to imply a univocality between media actors in this regard, or to impute a consistent intentionality on the part of journalists and editors. As will become evident through the example of *The Times*, sometimes its coverage was explicitly framed as filling a regulatory gap – but mostly its intentions were left unexpressed. Indeed, it may, at times, have amounted to little more than responding to commercial imperatives, providing information that it assumed would be of interest to its paying readership. From the decentred regulatory perspective of this article, however, discerning subjective intentions is less important than in exploring the potential effects of regulatory power as expressed through the various techniques adopted in relation to ‘informing’ its readership.

## Influencing Behaviour, Regulating CAM

### CAM's Normalisation by Biomedicine

The first regulatory technique highlighted that this once deviant set of healthcare practices, dismissed as the preserve of quacks, was increasingly recognised as a respectable, viable form of healthcare; one that the newspaper was apparently happy to cover in earnest, rather than ignore, decry or warn its readers away from wholesale. Yet, within this subset of stories, CAM's respectability was proportionate to its normalisation by the techniques, institutions and professions of biomedicine (Wahlberg, 2008). Thus, there were two primary means by which this process could be discerned in the data.

The first was evident in the multiple news stories concerning the emergence of objective (i.e. biomedical) proof that individual CAMs ‘worked’. CAMs, such as herbalism (The Times, 1983a; Woodham, 2003a), magnetism (Ahuja, 1997b) and acupuncture (Hawkes, 2004, 2007; Lister, 2009; Moody, 2016; The Times, 2013; Woodham, 2003b) were said to demonstrate potential biopolitical utility, as preliminary medical research or clinical trials indicated a potential efficacy in treating different conditions or associated symptoms. That clinical trials had been announced or were currently ongoing was the focus of a number of other articles, including less obviously biomedicalised modalities, such as faith healing (Ahuja, 1997a; Brompton, 1985; Timmins, 1985). Indeed, one article on faith healing from the 1980s observed that ‘decades of doubt and suspicion’ could be displaced through a ‘full-scale medically controlled research programme intended to produce irrefutable scientific proof’ that faith healing ‘works’ (Brompton, 1985). Implicitly, this form of normalisation required CAMs, which operated according to their own (perceived) deviant epistemologies and registers of health and illness, to be translated and proven according to the norms governing scientific, biomedical research. Sometimes this translation entailed an explicit dismissal of a therapy's own conceptions of healing and disease, such as in an article on acupuncture's evidence base, which informed readers that there was no anatomical evidence of ‘chi’ energy pathways – and that, according to experts, ‘the so-called meridians don’t really exist’ (Murcott, 2003).

The second means of biomedical normalisation was evident in news reports which suggested that the line separating biomedical practitioners and CAM practices was becoming porous, as biomedical institutions and practitioners began to provide CAMs to interested patients in greater numbers. This was evident in news stories covering specialist CAM medical facilities staffed by qualified medical doctors: for example, a story from the mid-1970s announced the opening of Britain's first hospital in ‘fringe medicine’, which was a private 80-bed facility staffed by ‘qualified medical practitioners’ that would deliver conventional care as well as homeopathy, acupuncture, osteopathy, nature cure and faith healing (Bailey, 1975). Other times, stories documented CAM therapies being used within ostensibly biomedical hospitals or clinics, such as in stories on acupuncture successfully being used during childbirth in biomedical hospitals (The Times, 1981). Furthermore, several articles throughout the dataset observed that general practitioners (GPs), in particular, were increasingly employing CAM therapies when dealing with patients, either training in CAM themselves or by referring patients onwards to undergo CAM therapies (Ingliss and West, 1984; Ive, 2004; McKee, 1991a; Smyth, 2018; The Times, 1983c, 1985). The link between the respectability of CAM and its uptake by biomedical professionals was explicitly outlined in one 1980s feature, which suggested that: ‘[a]fter years in a wilderness of suspicion and scepticism’ CAM was gaining ‘a healthy new respect’, as ‘many family doctors have already taken up one of these skills or decided you are a suitable case for treatment’ (Ingliss and West, 1985).

Hence, the first regulatory technique presented CAMs as a respectable choice to the newspaper's readership insofar as ‘objective’ testing, conducted according to the gold-standard of clinical trials, had demonstrated that a given therapy ‘worked’; and that a properly qualified medical doctor, trained in biomedical science, regarded such therapies, when performed by biomedical professionals, to be a useful tool of healing. Through this technique, the curious reader was implicitly and explicitly encouraged to try CAM within biomedical institutions, practiced safely by biomedical professionals, especially as there was seemingly an increasing amount of ‘proof’ that such therapies ‘worked’.

### Problematising Biomedicine, CAM as a Solution

At the same time that the newspaper was demonstrating the apparently successful biomedical normalisation of CAMs, a second, somewhat contradictory, regulatory technique could be discerned. This technique also presented CAM as a respectable and viable healthcare choice for the CAM-curious reader, but did so not through extolling CAM's efficacy as ‘proven’ through the adherence to the norms of scientific, biomedical testing or its uptake by scientifically minded, medically qualified doctors. Rather, it hinged upon CAM's fundamental difference to biomedicine, which, importantly, was itself problematised and to which CAM was posited as a solution (Ballantyne, 1993; Laurence, 1996; McGlashan, 1983; McKee, 1989a; No author, 1983). In other words, CAMs were respectable and had therapeutic value to the reader precisely because they resisted normalisation by biomedicine.

A number of stories made reference to CAM's purported natural, gentle and/or holistic approach, which was then contrasted with biomedicine's synthetic, harsh, and/or impersonal and mechanical way of healing. For example, in a series of features on CAM spread out over three consecutive days in August 1983, biomedicine was repeatedly critiqued for: its (in)efficacy with chronic illnesses (Ingliss and West, 1983b); its focus on powerful pharmaceutical interventions and concomitant iatrogenic illnesses (Ingliss and West, 1983a); and the ‘curative’ bias of biomedical practitioners (Ingliss and West, 1983c). At the same time, CAM was in each instance positioned as the solution to such problematisations, having apparently enjoyed success in treating chronic illnesses (unlike biomedicine), employing a wider repertoire of gentle therapeutics (unlike toxic pharmaceuticals or debilitating surgeries employed by biomedicine) and treating patients holistically (unlike biomedicine's impoverished mechanical view of the body). ‘[P]ractitioners of the once derided “fringe medicine” are in demand’, read one of the features, noting that 20 years ago CAM had seemingly been ‘swept aside by the triumphant march of medical science’ (Ingliss and West, 1983a). Yet, as the feature noted, disillusionment with scientific biomedicine and the toxicity of pharmaceuticals had seemingly ‘transformed’ CAM's ‘prospects’ (Ingliss and West, 1983a), with increased demand from patients as a result.

The idea that an overreliance in biomedicine on scientific norms and practices had given rise to a system of healing that was riddled with problems for the patient was echoed in an accompanying editorial. Rather than softening the problematisations of biomedicine to which CAM was presented as a solution, it instead underscored them, setting out the ‘disenchantment’ felt by many with ‘the purely scientific approach to medicine’ (The Times, 1983b). The editorial repeatedly juxtaposed the science of biomedicine (with medical doctors described as ‘dazzled by the objective, computerised approach to healing’ in which statistics ‘dominate’, reducing human beings to ‘groups of units’) and the alterity of CAM (which ‘looks at the completeness of an individual – physically and psychologically – and not just as the measurable facts of a physical condition’), finding biomedicine lacking and suggesting it had much to learn from CAM (The Times, 1983b). Noting that scientific research had ‘failed to provide satisfactory answers’ to a number of medical conditions, it concluded not only that science had not ‘earned the right to demand absolute conviction from possible patients’, but that biomedicine should recognise an ‘extra dimension to the art of healing’ which CAM could provide – and which biomedical science could not (The Times, 1983b).

Although the problematisation of biomedicine may have appeared incongruous when placed next to stories reporting on CAM's normalisation by biomedicine, the two techniques examined thus far operated in a similar fashion. *The Times* was, through such reporting, subtly opening CAMs up as viable, respectable, normal forms of healthcare to which the normal reader could turn. And, by reporting on its successes, whether vouched for by biomedical science or its professionals, or in terms of overcoming the problems generated by biomedicine, the newspaper presented it as a potentially wise choice for healthcare consumers; something they may want to consider when caring for their health or the health of their family.

### Highlighting CAM's ‘Risk’

A third regulatory technique evident in the newspaper concerned the dissemination of risks (Ewald, 1991). Understanding the link between risk and self-regulation of healthcare users requires an awareness of ‘the ways in which techniques and strategies that are developed to mitigate risk also work to harness the responsibilities and self-governing capacities of individuals to serve particular goals or ends’ (Wilkins and Gobby, 2022: 4). Articulating risks, then, is often linked to the articulation of strategies for individuals to mitigate risk: providing knowledge of what is ‘risky’ is a means of ‘empowering’ healthcare users, such that they can ‘choose between a rational course of action or other courses of action that [they] may still choose, *but at [their] own risk*’ (Greco, 2009: 20).

It was against broader Parliamentary interest, from the mid-1980s, in encouraging ‘better’ formal regulation amongst CAM practitioners that the articulation of risks in relation to CAM practice was particularly apparent in *The Times* (Wahlberg, 2015). It is notable that, if CAM practice was recognised as potentially ‘risky’, *The Times* did not call for direct governmental intervention through statutory regulation of healers. There appeared to be an assumption that CAM's ‘risks’ could be managed adequately without the need for inefficient, expensive, intensive state-centred forms of legal regulation (The Times, 1983b). Yet rather than leave readers to find their own way, *The Times* sometimes took it upon itself to provide the necessary guidance – and, in the process, acted to induce a particular type of responsibilised self-regulation on the part of the prospective CAM user.^2^

One element in inducing risk-mitigating self-regulation in the reader involved appealing to their emotions (Ashworth, 2017), instilling some measure of anxiety and vigilance through the small but rather powerful reminder that ‘anyone can set themselves up’ as a CAM therapist (Bueno, 2000; Naish, 2005; Ostrov, 1990; Stuttaford, 1996; Wright et al., 2003). The nature of the self-regulating field was such that there were few impediments preventing anyone – regardless of ability or experience – from ‘setting up shop’ as an acupuncturist (Prentice, 1990), aromatherapist (Murray, 1999), herbalist (Wright and Crompton, 2004) or (prior to 1993, when the profession gained statutory backing) osteopath (Fielding, 1988). Were CAM conceptualised as risk-free, the lack of regulatory impediments to operating as a CAM practitioner would not be problematised. However, the potential consequences such a situation generated for the user were spelled out at various points: homeopathy, when performed by ‘an ignorant practitioner’, was said to do ‘damage’ (McKee, 1989b); osteopathy, ‘in the hands of an unskilled practitioner’, was said to be potentially ‘dangerous’ (Purvis, 1989); while nearly all cases of ‘adverse effects’ from acupuncture were said to stem from ‘incompetent practice’ (Crompton, 2000). ‘Allowing an incompetent or a charlatan to dabble with your body can have serious consequences’, warned one feature, noting that ‘[m]ost alternative practitioners do not have standards they have to meet before being allowed to set up shop’ (Naish, 1998). The situation was described in hyperbolic terms by the BMA in one article, in which they suggested, given the light-touch regulatory regime that existed in the UK, that incompetent CAM therapists ‘would not get shut down until [they] had killed a few people’ (Jenkins, 1991).

Crucially, then, ‘risks’ mostly hinged on being attended by a ‘bad’ practitioner. When initial reports (debunked in the newspaper soon thereafter) emerged in 1990 that patients undergoing CAM with conventional treatments at the Bristol Cancer Hospital suffered higher death rates than those who opted solely for the latter (Sherman, 1990), *The Times* emphasised in an editorial the need to divide ‘the sheep from the goats’ in terms of healers (The Times, 1990). Moreover, the risks for the reader were occasionally linked to more than mere incompetency. For example, the front-page news that a hypnotherapist had been arrested for sexually assaulting 30 of his patients was tied to the question of regulation (Tendler, 1993): the inside story warned readers to ‘beware’ of ‘bogus hypnotherapists’ and included the prominent pull-quote, ‘There is very little control over who can set themselves up as complementary therapists’ (Landsdale, 1993).

Given the apparent risks to health and personal safety posed by ‘bad’ practitioners – whether negligent, poorly trained or criminal – readers were to be vigilant when selecting a therapist and making ‘responsible’ choices in this regard was crucial. Thus, the newspaper at times explicitly sought to provide information to help readers take ‘the guesswork out of finding the best practitioner’ (McKee, 1989b). For example, an in-depth series of lifestyle features over five consecutive days explored the most popular CAM therapies, including homeopathy, acupuncture, and chiropractic, as well as range of what it termed ‘new age’ therapies, such as crystal healing. In each case, the reader was consistently guided towards contacting formal regulatory bodies, such as professional associations and training schools, including institutions such as the Society of Homeopaths, that were said to represent or produce qualified, competent practitioners in each particular field. Simply ensuring that one's healer belonged to a professional organisation was not enough to dispel the necessity of continuous vigilance in the CAM user. ‘[P]roceed with caution’, warned one feature, addressing the potential CAM user directly: ‘check that the practitioner you choose has been trained and registered, if possible, with a reputable organisation’ (McKee, 1991b).

Hence, the vigilance induced in the potential CAM user extended into ascertaining whether or not the body in question was ‘reputable’ and therefore associated with ‘good’ practice. In keeping with this distinction between ‘good’ regulatory bodies and ‘bad’, several articles and features problematised, in some capacity, the fragmented regulatory field, which was itself generative of hazards and risks associated with poor practice, as it failed to adequately triage out ‘bad’ practitioners (McKee, 1989b). For example, one highlighted how standards between schools ‘vary tremendously’ and that people often had to take it on ‘trust’ that ‘the person who is sticking needles into them has been properly trained’ (Gomer, 1991), while another focussed on the sheer volume of different bodies: 143 professional organisations, covering 14 disciplines, with at least 100 training courses for different CAM therapies, many of which were unaccredited by the organisations concerned (Murray, 1997).

One of the striking aspects to its coverage is that while *The Times* sometimes cultivated anxieties directed towards to the figure of ‘poorly’ trained, negligent or criminal CAM practitioner (Doel and Segrott, 2003a), it often seemed to place the responsibility for patient safety not on formal regulatory bodies or the national government – but on the prospective CAM user themselves. Readers were enjoined to ensure that the therapist in question was ‘adequately’ trained and qualified and were consequently guided towards contacting professional associations for lists of registered practitioners. *The Times* provided the tools by which potential CAM users could be ‘empowered’ into navigating the fragmented regulatory field as it actually existed, by calling on readers to ‘do their homework’ in conjunction with the information contained in the newspaper (Woodham, 2000).

### Auditing CAM

A fourth regulatory technique concerned the employment of audit, as described by Power (Power, 1997); a technique which features in decentred healthcare regulatory literatures (Trubek et al., 2008). Given that audit had become generalised across multiple domains of social existence in the UK by the 1980s and 1990s, it is perhaps unsurprising that even newspapers began to display their own will to audit, something which was evident not only in relation to CAM, but across the various rankings, league tables and guides on universities and graduate employment, and so on.^3^ The application of audit to CAM was not limited to newspapers: Wahlberg, for example, characterises Parliament's indirect attempts to help CAM practitioners ‘get their own house in order’ from the 1980s onwards as an attempt to make CAM ‘auditable’ (Wahlberg, 2015).

Audit regulates by providing a ‘versatile means of purporting to render accountable and judgeable the activities’ of almost anyone and anything (Rose, 1996: 351). It translates rarefied silos of substantive knowledge otherwise inaccessible to outsiders, such as medicine, into a common technical language; a process by which ‘complicated contextually variable phenomena’ can be transformed into ‘unambiguous, clear, and impersonal measures’ that render disparate objects comparable to each other and against an idealised benchmark (Merry, 2011: 584). Although such techniques render diverse domains amenable to external oversight, more important for our purposes is that once audits are made publicly available, such as through a newspaper, they can also be used to nudge, guide or otherwise inform choice in a given domain.

As has been outlined thus far, normalisation by biomedicine was a technique which resulted in clinical trial results being reported across the newspaper. Similarly, warnings of the necessity of ‘doing one's homework’ regarding healers and their self-regulating bodies were also a feature of *The Times*’ CAM coverage. The use of audit, however, provided an intensification; a more efficient means of re-representing such issues (and, consequently, of guiding potential CAM users), sometimes appearing as a small part of a single feature, and sometimes as a multi-page, multi-edition supplement, framed as A-to-Z guides to CAM, to be retained by readers as an authoritative future reference point on the subject.

The first kind of audit was directed to CAM therapies: techniques were employed to assess the quantity and quality of scientific evidence underpinning them and provided the basis for ranking therapies against one another (Ahuja, 2006; Naish, 2005; The Times, 1996, 1998a, 1998b, 1998c, 1998d, 1998e, 1998f). Results were presented in a table or bar-chart format, with the highest scoring therapies (and therefore those with the ‘strongest’ evidence) at the top, and the rest listed in descending order, with the therapies underpinned by the qualitatively and quantitively weakest evidence at the bottom. While some therapies, such as acupuncture, were ranked consistently near the top of the score board, others, such as homeopathy, had shifting fortunes, moving from high to low positions.

In addition to the evidentiary audits, a second kind of audit could be discerned, which was directed towards CAM practitioners themselves. A one-off *Times’ Complementary Therapists Guide* – a comprehensive 138-page supplement – was published in two parts in 2004 and aimed to audit the standards of CAM practitioners working in what House of Lords in 2000 called the ‘big five’ (acupuncture, herbalism, homeopathy, chiropractic and osteopathy). A preface by editor Hilly Janes was explicit that the guide was filling a regulatory ‘gap’ – and that it would help readers in understanding how to ‘choose wisely’ when selecting a therapist; that is, to separate out the ‘properly qualified, accountable and ethical professional’ CAM therapist from the ‘fly-by-night chancer with only a consulting room and a brass plate to his or her name’ (Janes, 2004b: 3). It is particularly notable, then, that at the time of its publication, osteopathy and chiropractic had been subject to SSR for over a decade, something which coverage had generally regarded as a positive development and which the guide itself described as ‘good news’ (Janes, 2004a: 3). Nevertheless, that statute underpinned the self-regulation of such professionals was evidently not itself a guarantee that such practices were safe for users. Thus, the guide functioned as a directory, with individual therapists for each of the ‘big five’ therapies listed alphabetically by geographical region, each with a corresponding set of symbols to denote their adherence to a set of best practice criteria developed by *The Times*. These included standards about information provided to patients, stopping ineffective treatment, communicating with a patient's GP, continuing professional development and the taking of patient history. Failure on any of the metrics would result in the absence of the corresponding symbol from a practitioner's name, such that readers would be able to distinguish therapists with top scores across all best practice criteria within their locale – and, conversely, those therapists who were deficient across one or more metrics (The Times, 2004).

Rather than scattered news articles covering discrete clinical trial successes or features recommending particular schools of regulatory bodies, the use of audit techniques in both cases served as a more comprehensive means of guiding the prospective CAM user, helping them once again to make ‘informed choices’ in the potentially limitless marketplace of CAM. Audit techniques such as ranking and rating operate as ‘subtle regulatory tools’ (Cooley, 2015: 18), translating the results of complex and inaccessible research (which the average reader would be unable or unwilling to assess themselves) into simple, easy to follow graphics and scores. Readers could be guided towards those therapies for which there existed the strongest evidence or towards therapists who demonstrated the highest standards and, correspondingly, nudged away from those for which there was weaker evidence or therapists which were found wanting on one or more metrics of practice.

### (Re)problematising CAMs

The final regulatory technique concerned the re*-*problematisation of CAMs on the basis of a failure of biomedical normalisation. Whilst this technique was employed to varying degrees more or less throughout the dataset, it appeared particularly intense in the final decade of the coverage, as a concerted pushback against CAM's funding in the NHS, its teaching at universities, and its presence in other public institutions, such as the Charity Commission, was underway by the 2010s, led by a coalition of anti-pseudoscience campaigners (Caldwell, 2017).

Importantly, the technique of (re)problematisation was frequently limited in its scope, signalling not that CAM as a whole or even a given form of CAM, such as acupuncture, was to be rejected outright, but rather that, for a given condition, biomedical testing had yielded mixed results or no proof of healing effects beyond that of a placebo. Thus, acupuncture was described as ‘failing smokers’, on the basis of a meta-analysis of 20 years’ worth of clinical trials on the subject on treating nicotine addiction (The Times, 2000), although acupuncture itself was not dismissed wholesale. Elsewhere, in a discussion of treatments for allergies, readers were advised that while some clinical trials had demonstrated acupuncture's success, the most recent clinical trial data demonstrated no evidence of acupuncture's efficacy. However, rather than necessarily caution readers or warn them away, the article concluded that ‘more research is needed’ (Murcott, 2005).

However, at other times, entire CAM therapies, such as chiropractic or homeopathy, came close to being written off almost entirely on the basis of meta-analyses which were interpreted as demonstrating their limited therapeutic value *per se*. Chiropractic, for example, which was at various points held up as a biomedically valid therapy for conditions such as back pain, was described in one headline from 2006 as providing a ‘worthless form of treatment’ (Hawkes, 2006). Sometimes this dismissal of entire CAM therapies was less reactive (i.e. on the basis of recent clinical trial failures) and more related to what was perceived as the irredeemably deviant nature of a therapy's underlying beliefs and practices, which were incorrigible or fundamentally irreconcilable with biomedicine. It was in these data, largely emanating from columnists, rather than hard news articles, that an outright sarcastic, scornful or mocking tone was sometimes used, with the presumable intention of underscoring that a given CAM therapy was a laughing stock, rather than a respectable form of healing (Caldwell, 2017). For example, homeopathic medicines, which operate on the principle that the more diluted the medicine, the more potent the healing effects, were described as ‘sugar pills’ and homeopaths as ‘water sellers’ by some columnists, alluding to the fact that such medicines are diluted to such a degree there is often, from a pharmacological perspective, no active ingredient remaining (Pollard, 2014). At other points, the irredeemable deviance of a given CAM practice was problematised through unflattering comparisons with other unproven and implausible ideas that were beyond the pale for rational, scientific biomedicine. For example, Simon Singh, head of the anti-pseudoscience pressure group Good Thinking Society, authored a column in which he ridiculed homeopathy by analogy with conspiracy theories: ‘I will not outline the vast body of scientific evidence that shows that homeopathy is ineffective’, he wrote, ‘just as I am not going to explain why … the Queen is not an alien lizard’ (Singh, 2015).

Finally, CAMs were also occasionally problematised on the basis of their apparent dangerousness, which – unlike the risks identified in relation to ‘poor practice’ across multiple CAM therapies – could not be mitigated by doing's one's homework and ensuring one went to a properly regulated healer. At times, this related to particular products or practices which were simply dangerous in and of themselves – such as specific herbal teas which were linked to liver failure (Gee, 1989; Hawkes, 1994; Murphy, 1993), or the practice of vitamin megadosing, which was said to have left individuals disabled (Wright, 1983). However, even practices which were at times presented as risky solely in the ‘wrong’ hands were, at other times, presented as dangerous *per se*. Thus, in the wake of homeopathy being defunded from the NHS and amidst a backdrop of intense campaigning by anti-pseudoscience figures (Caldwell, 2017), the PSA's decision to reaccredit the Society of Homeopaths (the largest representative body for non-medically qualified homeopaths in the UK) received critical coverage in the newspaper. In an editorial which ran under the subtitle ‘Homeopaths can cause great harm and deserve to be stripped of their accreditation’ (The Times, 2019), the newspaper repeatedly presented homeopathy as dangerous: its usage by consumers could cause ‘fatal delays’ in receiving ‘proper’ (biomedical) treatment and, even where its effects were not fatal, it could ‘make sick people sicker’ (The Times, 2019). Perhaps most notably, homeopathy was said to overlap ‘with dangerous beliefs’ (i.e. beliefs which directly contradict biomedical science and public health policies) such as the anti-vaccination movement – and it was therefore ‘important to fight this dangerous belief system’ (The Times, 2019). Although the PSA was designed to combat poorly trained, poorly regulated healers by providing a state-sanctioned accreditation scheme to certify their standards of practice, this was irrelevant in the face of the dangers homeopathy apparently posed to prospective users. In other words, good and bad homeopathic practitioners could not be distinguished on the basis of training, qualifications and other facets of professional competency: there were no ‘good’ homeopaths of which to speak – meaning the PSA's decision would do little to keep people safe, but may place people at risk by providing a veneer of respectability to (what was problematised as) a fundamentally deviant and dangerous form of quackery.

## Conclusion

In this article, I identified five techniques – normalisation by biomedicine, problematisation of biomedicine, risk, audit, and (re)problematisation of CAMs – through which *The Times* newspaper was engaged in decentred regulation. These techniques operated, in their own ways, by appealing to readers’ freedom to choose in the market-place of CAM. They assumed and encouraged a particular form of rational, responsible, self-regulation, in which prospective users would opt for ‘safe’ and/or ‘effective’ healers and healing practices (however safety and efficacy are conceptualised) – and, correspondingly, avoid those problematised as risky, ineffective and/or otherwise deviant. In other words, the coverage seemingly shared some of functions of formal healthcare regulatory bodies, albeit through enlisting (or attempting to enlist) prospective CAM users to keep themselves safe, ensure they accessed ‘quality’ healing practices that ‘work’ and were seen by competent, qualified, and well-regulated professionals. The line separating those therapies which ‘worked’ from those which did not, those to which the medical profession was opposed or in favour, those which were deemed safe (in the right hands) or risky (*per se*) was far from fixed. Therapies conceptualised as efficacious for a given purpose across one set of articles may, in subsequent stories, have been dismissed wholesale or in part as ineffective quackery. Yet where such lines were drawn (and how those lines were redrawn) over the years is less important from the perspective of this article than the fact a line was drawn at all for readers; that is, that prospective CAM users could be guided towards or away from therapies by the news media on the basis of their purported safety, efficacy, quality and so on.

Moreover, one of the striking aspects of the coverage was that the purported quality of the formal self-regulatory regime of CAM professionals was itself a basis upon which users were encouraged towards or away from particular CAMs and their professionals. In a kind of informal meta-regulation, readers were shepherded towards various ‘reputable’ professional associations governing CAMs and, in the case of the directory-style audit of the ‘big five’ therapies, towards individual healers. It is particularly notable that while the coverage generally framed SSR for chiropractic and osteopathy as a positive step, that statute underpinned their formal regulation was not necessarily a guarantee such practices were ‘safe’ – and users were therefore encouraged to remain vigilant. Thus, regardless of whether a given CAM user was to be attended by a self-regulating or SSR CAM professional, readers were frequently guided through the complex formal regulatory space of CAM, provided with questions to ask, clues to look for, signs and symbols to understand that may help to separate out the qualified from the qualified, the quack from the genuine healer.

It might be said that if newspapers have been able to provide such regulatory direction in relation to CAM users, it is a consequence of the state's uniquely hands-off approach to CAM's regulation and provision, where centralised systems of information for health consumers are generally lacking and where a private market of providers predominates. In contrast to biomedicine, whose practitioners are regulated by the GMC, which is accessed by most Britons through the NHS, and where patients consequently enjoy comparatively limited choice, prospective users entering the marketplace of CAM are obliged to choose: to determine the therapy, the healer, and which register or professional association to turn. Yet, choice is far from absent from biomedical healthcare in the UK. Not only is there a private market of health services and products, with varying degrees of formal regulation which are frequently written about in the popular press (indeed, *The Times* launched its own ‘good doctor’ guide for private practitioners of biomedicine in 2002), but the language of choice has increasingly entered into the NHS itself, in the wake of successive neoliberal reforms since the 1980s.

Lastly, this article has focused on decentred regulation through the news media of a relatively marginal set of healthcare practices as compared to biomedicine. Yet the stakes of interrogating such everyday forms of healthcare regulation can be made even more apparent when considered against the backdrop of the COVID-19 pandemic. Print and broadcast news, long criticised in academic literature for misrepresenting science and medicine (Boyce, 2007), nevertheless formed an institutionally authorised source of information as the pandemic played out – that is, information produced by professional journalists and editors, using ‘official’ statistics or other ‘expert’, biomedical knowledges. In an era of social media, however, such knowledges compete with non-official sources of information that can spread just as quickly and as widely. Thus, UK print and broadcast news coverage during the pandemic more-or-less reflected the mainstream knowledge claims of state regulatory bodies, highlighting, for example, the safety and efficacy of newly created vaccines. On social media, these claims came under sustained attack from multiple angles, as competing sources of information not only problematised the safety, efficacy and/or quality of the vaccine, but promoted alternative forms of treatment which were said to be safe and/or efficacious, ranging from off-label use of ostensibly biomedical treatments, such as hydrochloroquine and ivermectin, to a number of different ‘unproven’ CAM treatments, which were said to provide safe and natural forms of protection against COVID-19. Hence, the stakes of interrogating the news media as a site of decentred regulation appear heightened where such regulatory techniques work to problematise or significantly undermine the conceptions of safety, efficacy and quality embedded within formal state regulatory regimes. Indeed, owing to its everyday nature, the news media – whether print, broadcast, digital or social – as a contested terrain of decentred regulation may, in fact, be just as influential in guiding prospective users towards or away from particular CAMs or other therapies on the basis of their purported safety, quality and/or efficacy as the more formal kinds of regulation typically examined in regulatory literatures.

## Notes

1. The exceptions are osteopathy and chiropractic, whose practitioners sought and were granted statutory self-regulation as independent professions in the early 1990s.

2. The ‘ultimate guide to CAM’ type feature can be seen across multiple print media outlets. See, for example, The Independent's eight-part weekly guide to CAM therapies (Ernst, 1998) which included assessments of risk for each therapy, The Observer Magazine's weekly A-to-Z of CAMs including Chinese medicine (Ke, 1991) or the Daily Mail's recent guides to different CAMs (Fleming, 2013).

3. While *The Times* was and is perhaps the most audit-friendly newspaper in general, other newspapers including the Daily Mail (Ernst and Singh, 2008), The Guardian/Observer (Jones, 1995) and the Independent (Ernst, 1998) applied audit techniques and ranking to CAM.
